# Goal Setting During Telerehabilitation in Japan: Qualitative Study of Occupational Therapists’ and Physical Therapists’ Experiences Using Reflexive Thematic Analysis

**DOI:** 10.2196/101355

**Published:** 2026-08-21

**Authors:** Yuho Okita, Kounosuke Tomori, Minoru Okita

**Affiliations:** 1 School of Health Sciences, Swinburne University of Technology, John Street, Hawthorn, Victoria, 3122, Australia, 61 3 9214 8000; 2 Department of Physical Therapy Science, Nagasaki University Graduate School of Biomedical Sciences, Nagasaki, Japan; 3 School of Health Sciences, Department of Rehabilitation, Major of Occupational Therapy, Tokyo University of Technology, Tokyo, Japan

**Keywords:** telerehabilitation, goal setting, reflexive thematic analysis, occupational therapy, physical therapy

## Abstract

**Background:**

Telerehabilitation expanded rapidly during the COVID-19 pandemic and is increasingly used alongside, or as an alternative to, in-person rehabilitation. Goal setting is a core component of client-centered rehabilitation; however, how therapists conduct goal setting remotely remains poorly understood, particularly in East Asian contexts. To our knowledge, no qualitative study has specifically examined remote goal setting in telerehabilitation in Japan, where remote delivery was not yet routine when these data were collected.

**Objective:**

This qualitative study aimed to explore how Japanese occupational therapists and physical therapists experienced negotiating and adapting rehabilitation goals during telerehabilitation, including how remote delivery shaped assessment, collaboration, and the purposes assigned to goals.

**Methods:**

Individual semistructured interviews (18‐51 minutes; mean 32 minutes) were conducted via Zoom (Zoom Communications, Inc) in Japanese with 9 therapists (7 occupational therapists and 2 physical therapists; 7 male and 2 female; aged 29‐43 years) who had direct experience of remote goal setting. Participants were recruited by purposive and snowball sampling from 9 different workplaces across Japan, including metropolitan areas such as Tokyo and regional areas such as Hokkaido and Nagasaki, and worked in hospital, private-practice, community-based, and academic settings. Data were analyzed using reflexive thematic analysis within a contextualist epistemology, following Braun and Clarke. Trustworthiness was considered using Nowell and colleagues’ criteria, and reporting followed the COREQ (Consolidated Criteria for Reporting Qualitative Research) checklist.

**Results:**

Four themes were constructed: (1) losing touch: uncertainty in remote assessment and goal setting (9/9 participants); (2) from direct intervention to coaching-oriented goal setting (8/9 participants); (3) at home, at ease: needs that surface in clients’ own environments (6/9 participants); and (4) when continuity becomes the goal (6/9 participants). Therapists described remote goal setting as shaped by both constraint and possibility. Partial bodily, nonverbal, and contextual information reduced their confidence in assessment and goal negotiation, and they responded through preparation, visual resources, self-monitoring tools, and collaboration with clients and families. Sessions conducted in clients’ own environments appeared to support comfort and sometimes made everyday needs more visible. When in-person care was unavailable or insufficient, therapists sometimes broadened goals from functional improvement alone to include continuity, ongoing monitoring, and prevention of disengagement.

**Conclusions:**

In these therapists’ accounts, remote goal setting was not simply a diminished version of in-person practice but a distinct clinical process requiring deliberate preparation, collaborative communication, and management of uncertainty. Hybrid pathways may combine in-person assessment, when hands-on examination is needed, with continued remote negotiation, monitoring, and revision of goals. Further research should incorporate clients’ and families’ perspectives and evaluate hybrid models of goal setting across diverse rehabilitation populations.

## Introduction

### Telerehabilitation and Remote Goal Setting

Telerehabilitation, defined as the delivery of rehabilitation services through information and communication technologies, such as videoconferencing, mobile apps, sensors, and telephone-based communication, has expanded rapidly in recent years [1,2]. This growth was particularly accelerated by the COVID-19 pandemic, which increased the uptake of remote health care across rehabilitation settings [3]. This expansion has created new opportunities to improve access to care and support continuity of rehabilitation services, particularly for people who face barriers to attending in-person appointments [4]. In addition to improving accessibility, telerehabilitation can offer other benefits, including greater convenience, reduced travel-related costs, improved adherence, and enhanced continuity of care [5]. Recent evidence indicates that telerehabilitation may have a positive role in supporting physical activity and functional outcomes among older adults. For example, a systematic review and meta-analysis of telerehabilitation-delivered exercise interventions for older adults with frailty, cognitive impairment, or mobility disability reported improvements in mobility, muscle strength, and balance [6]. Similarly, a recent meta-analysis of digital interventions for frail older adults found reductions in frailty scores and improvements in grip strength and health-related quality of life [7]. This evidence suggests that telerehabilitation may be a useful alternative or complement to in-person rehabilitation for diverse populations, including frail older adults and others with limited mobility [1,8]. However, its implementation remains constrained by several challenges, including limited digital literacy, difficulties in conducting physical assessments remotely, and insufficient training opportunities for clinicians [3]. Because telerehabilitation was initially adopted rapidly in response to urgent service needs, evidence regarding how to deliver client-centered rehabilitation in remote contexts remains limited [3].

Among the core processes of client-centered rehabilitation, reaching an agreement on goals and tasks is a central element of the therapeutic alliance that requires complex adaptations during the transition from in-person to remote service delivery [9]. Both client-centeredness and the therapeutic alliance are considered beneficial for rehabilitation: a stronger therapist-patient alliance has been associated with better outcomes in physical rehabilitation settings, including greater treatment adherence and satisfaction, improved physical function, and reduced depressive symptoms [10], and goal setting aligned with clients’ own priorities may improve engagement, motivation, and psychosocial outcomes such as self-efficacy [11]. Goal setting is widely regarded as essential to client-centered practice [11]. It helps establish priorities, guide intervention, and align rehabilitation efforts with what matters most to clients in their everyday lives [12,13]. In principle, rehabilitation goal setting should be client-centered and involve shared decision-making; however, previous research suggests that this is not always achieved consistently in routine practice [13,14]. When therapists and clients meet in person, goal setting is typically informed by clinical observation, physical assessment, and nonverbal communication [15], and is supported by embodied interaction where meaning and goals are often co-constructed and confirmed through physical guidance and felt bodily changes [16]. In telerehabilitation, these elements are constrained, as physical distance and technology alter nonverbal communication and eliminate hands-on interaction. Although technology may support goal setting through functions such as monitoring, feedback, and communication, this does not in itself explain how goal setting is actually carried out in remote rehabilitation encounters [12,17]. A recent scoping review identified a range of goal-setting tools used in adult rehabilitation, yet highlighted limited evidence regarding their design or validation for remote use [12]. Another scoping review focusing specifically on telerehabilitation and goal setting identified 68 studies, but none were conducted in East Asian countries, including Japan [17]. Goal setting and shared decision-making within remotely delivered rehabilitation have begun to attract attention elsewhere, for example, among older adults starting mobile health cardiac rehabilitation in the United States [18] and in a trial of decision aid-supported shared decision-making within hybrid in-person and virtual pulmonary rehabilitation in China [19]; however, no qualitative study has examined therapists’ experiences of goal setting in telerehabilitation in East Asia, including Japan. Examining how goal setting is experienced in telerehabilitation is therefore important, particularly in settings where it is not yet routine, as the challenges and adaptations of early implementation can become less visible once remote practice is normalized and embedded within established workflows.

### Japanese Rehabilitation Context

In Japan, goal setting in rehabilitation continues to be strongly influenced by traditional medical-model and clinician-led approaches [20]. This has prompted ongoing discussion about how goal-setting practices can become more collaborative and client-centered [14]. A cross-sectional study involving clients and occupational therapists in Japan found that, although both groups reported high levels of client engagement in the goal-setting process, nearly 80% of goal statements were mismatched between occupational therapists and their clients [13]. This finding suggests that even when both parties perceive goal setting as collaborative, their understandings of the goals may not necessarily be aligned, highlighting the need for higher-quality goal-setting practices [13]. This may also reflect culturally specific factors, as rehabilitation in Japan still tends to focus predominantly on functional training rather than activity- and participation-level goals aligned with what is personally meaningful to the individual [21]. Consistent with this, a recent qualitative study on goal setting in home rehabilitation in Japan highlighted the need for more explicit explanation and more collaborative approaches tailored to the Japanese context [21].

Japan’s rapidly aging population and geographic challenges point to the potential value of telerehabilitation for improving access to rehabilitation, particularly for people with mobility limitations, including frail older adults [1,22]. Regional disparities in rehabilitation provision continue to create uneven access across the country [23-25], and in this context, telerehabilitation may be particularly relevant. Recent studies have reported improvements in balance, gait, and lower-limb strength following remotely delivered exercise programs for pre-frail and frail older adults [26], and a pilot study in Japan found improvements in health-related quality of life after a multiuser telerehabilitation exercise intervention for older adults with frailty [27]. However, the uptake of telerehabilitation in Japan remains limited compared with some other countries, partly because of regulatory and reimbursement constraints, and remote rehabilitation may represent a particularly significant shift in practice for Japanese therapists [20,28,29]. At the time of this study, telerehabilitation in Japan was not yet routine practice: it was typically introduced when in-person care was difficult or impossible, for example, during the COVID-19 pandemic, after hospital discharge, or in geographically underserved areas, and it was largely delivered outside standard reimbursement schemes [28,29]. The experiences examined in this study should therefore be understood against this backdrop of early, often improvised, adoption.

### Study Aim

Considering these contextual factors, it is important to understand how therapists in Japan experience the delivery of telerehabilitation, including the goal-setting process. Such knowledge may help identify research priorities, inform clinical training, and support policy discussions on reimbursement and service delivery. Because telerehabilitation was not yet routine in Japan when these data were collected, therapists’ accounts of this period can document challenges and adaptations that may become less visible once remote practice is normalized. Therefore, this qualitative study aimed to explore how Japanese occupational therapists and physical therapists experienced negotiating and adapting rehabilitation goals when delivering telerehabilitation across a range of clinical settings in Japan, including how remote delivery shaped assessment, collaboration, and the purposes assigned to goals. Because goal setting is embedded within the broader flow of remote practice, therapists’ wider experiences of telerehabilitation were also explored to the extent that they shaped how goals were identified, negotiated, and followed up. To obtain a broad understanding that can inform future research, participants were not limited by practice setting or target population.

## Methods

### Study Design

This study used a qualitative descriptive design informed by reflexive thematic analysis (RTA), following the 6-phase approach articulated by Braun and Clarke [30,31]. Consistent with RTA, the study was positioned within a contextualist epistemology, which acknowledges that participants’ accounts reflect situated experience shaped by cultural, institutional, and interpersonal contexts, and treats the researcher as the analytic instrument through which meaning is constructed rather than discovered [30-32]. Reporting was guided by the COREQ (Consolidated Criteria for Reporting Qualitative Research) checklist (Checklist 1) [33]. A reflexive thematic approach was selected because the study aimed to explore the experiences, perceptions, and meanings that therapists ascribed to remote goal setting in rehabilitation practice.

### Ethical Considerations

Ethical approval for this study was obtained from the Ethics Committee of the Graduate School of Biomedical Sciences (Health Sciences), Nagasaki University (approval number: 22041401). All participants provided written informed consent before participation: each participant received a written information sheet by email as part of the study invitation (see Participants and Recruitment), and signed consent forms were returned by post, with the original forms stored securely at the second author’s institution. Participants were informed that participation was entirely voluntary and that consent could be withdrawn at any time without disadvantage. Participants did not receive financial or other compensation for taking part in this study. Interviews were conducted in Japanese and transcribed verbatim. Participant anonymity was protected through the use of identification codes, and all direct identifiers were removed from transcripts. Video recordings and study data were stored securely and will be retained for 5 years in accordance with institutional policy.

### Research Team and Reflexivity

The research team comprised 3 authors. The first author (YO) is a male occupational therapist holding BExSc(Hons) and MOT (Master of Occupational Therapy) qualifications at the time of the study, with clinical experience in Australia and formal training in qualitative research through doctoral study; YO conducted all interviews and led the analysis. YO had a research interest in client-centered practice and telerehabilitation prior to the study and approached telerehabilitation as promising but underevidenced in Japanese practice. The second author (KT) is an occupational therapist and university-based researcher with expertise in collaborative goal-setting tools and client-centered practice in Japan; KT contributed to study design, interview guide development, and analytic oversight. The third author (MO) is a physical therapist and university-based researcher with expertise in rehabilitation science in Japan; MO contributed to study design, participant recruitment, and analytic supervision. None of the authors had a prior clinical relationship with the participants at the time of interview; however, several participants were known to the authors through prior professional contact or professional-network introductions, and this was disclosed and discussed in team meetings as part of reflexivity. An audit trail of the sequential analytic process was maintained in a separate document, documenting initial analysis, integration of initial analysis, initial theme generation, and final theme development with illustrative quotes [34]. These materials were shared with all authors for review and discussion [34,35]. Consistent with RTA, interpretation was recognized as shaped by researcher subjectivity and not expected to be identical across researchers; team discussions were used to challenge interpretations, refine thematic boundaries, and strengthen the coherence, credibility, and acceptability of the final thematic structure [35,36].

### Participants and Recruitment

Participants were recruited using purposive and snowball sampling to identify therapists with direct experience of remote goal setting in rehabilitation practice [37,38]. For the purposes of this study, remote goal setting was defined broadly to include remote discussions of clients’ needs, priorities, and goals that informed rehabilitation planning. Licensed physical therapists and occupational therapists practicing in Japan who had experience with remote goal setting were eligible to participate. No additional exclusion criteria were applied.

Potential participants were identified through the professional networks of the research team and through participant referral. Each potential participant was contacted individually by email with an explanation of the study purpose and an invitation to take part. Each participant worked at a different organization; no two participants were employed at the same workplace at the time of the interview. This was a deliberate consequence of the sampling strategy and reduces the likelihood that the themes constructed reflect a single institutional culture. Participants were drawn from both metropolitan areas (eg, Tokyo) and regional areas of Japan (including Hokkaido and Nagasaki) and worked across a variety of settings, including hospital-based services, private practices, and community-based services. Information on participants’ clinical setting and the geographic location of their practice was identified from interview content during the familiarization phase of analysis and recorded in the audit trail; this level of demographic information was not captured in the structured online questionnaire. For one participant (Participant 8), the region of practice could not be confirmed from these sources and is therefore not reported in Table 1.

**Table 1.** Participant characteristics (N=9)^a^.

ID	Profession; sex; age (years)	Experience, years (remote)	Remote practice context	Region
P1	OT; male; 34	12 (<1)	Adults (19-65 years); hospital; neuromuscular disorders	Kanto
P2	OT; male; 38	16 (1)	Children (0‐18 years); community; neurodevelopmental disorders	Hokkaido
P3	PT; male; 29	8 (6)	Adults (19-65 years); private practice; musculoskeletal conditions	Kanto
P4	PT; male; 42	21 (1)	Older adults (65+ years); hospital; musculoskeletal conditions	Kyushu
P5	OT; male; 39	17 (7^b^)	Adults (19-65 years); community; psychosocial disabilities	Kansai
P6	OT; male; 37	14 (3)	Children (0‐18 years); academic; neurodevelopmental disorders	Chubu
P7	OT; female; 41	19 (1)	Older adults (65+ years); hospital; musculoskeletal and neuromuscular conditions	Hokkaido
P8	OT; male; 43	19 (1)	Children (0‐18 years); community; musculoskeletal and neuromuscular conditions	Not reported^c^
P9	OT; female; 31	12 (1)	Children (0‐18 years); community; neurodevelopmental disorders	Kanto

a OT: occupational therapist; PT: physical therapist. Experience is reported as total years of therapist experience, with years of remote service experience in parentheses. Region indicates the broad geographic region of Japan in which the participant practiced, reported at this level to protect participant anonymity: Kanto (eastern Honshu, including the Greater Tokyo area), Chubu (central Honshu), Kansai (western Honshu, including Osaka and Kyoto), Kyushu (the southwestern main island, including Nagasaki), and Hokkaido (the northernmost main island).

b Approximately 7 years, with infrequent use.

c The region of practice for this participant could not be confirmed from the interview or questionnaire data and is therefore not reported.

Sample size was guided by the concept of information power rather than by a predetermined target or data saturation [39]. Applying Malterud and colleagues’ [39] five criteria, we judged that the sample could support the study aim on the basis of (1) a narrow and focused aim (therapists’ experiences of remote goal setting); (2) dense specificity of participants (licensed, experienced therapists with direct remote goal-setting experience, drawn from 9 workplaces across metropolitan and regional Japan and a variety of clinical settings); (3) an established conceptual grounding in client-centered goal setting; (4) focused semistructured interviews conducted by a trained qualitative interviewer using a flexibly applied topic guide (see Data Collection); and (5) a cross-case, theme-oriented analytic strategy. Information power offers a structured basis for judgment rather than a guarantee of completeness, and the uncertainty inherent in this judgment is acknowledged in the Strengths and Limitations section.

### Participant Characteristics

Nine therapists participated in the study, including 7 occupational therapists and 2 physical therapists. Of these, 7 were male and 2 were female. Participant ages ranged from 29 to 43 years, and years of therapist experience ranged from 8 to 21 years. Experience with remote service delivery ranged from less than 1 year to approximately 7 years. Participants had experience providing remote services to children, adults, and older adults across a range of clinical areas, including neurodevelopmental disorders in children, musculoskeletal disorders or injuries, neuromuscular disorders, and other conditions. Detailed participant characteristics are presented in Table 1.

Participants delivered remote services mainly by videoconference. Consistent with the early-adoption context described in the Introduction, they had typically introduced remote delivery when in-person care was difficult or impossible, for example, during pandemic-related service disruption, after hospital discharge, or for clients in geographically underserved areas. The frequency and duration of remote sessions were not collected systematically in the questionnaire; the following patterns were identified from interview content during the familiarization phase. Remote service formats were heterogeneous: they ranged from brief, repeated contacts of about 10-30 minutes to sessions of about an hour, delivered at rhythms from weekly to monthly, and several participants combined scheduled video sessions with asynchronous contact between sessions, such as email, messaging applications, activity diaries, and videos recorded and submitted by family members. Examples included weekly 30-40-minute videoconference sessions continuing a hospital program after discharge; a 3-month package of 5 video consultations (an initial 40-minute goal-setting session followed by 20-minute follow-ups) supplemented by unrestricted text chat; and regular 15‐30-minute video calls that complemented, rather than replaced, in-person visits. Because no standardized comparison with usual in-person schedules was collected, the study does not infer that remote delivery itself shortened sessions or changed their frequency.

### Data Collection

Semistructured interviews were conducted individually via Zoom videoconference between September and October 2022. Interviews lasted between 18 and 51 minutes, with a mean duration of 32 minutes. Zoom was selected for its ease of use, cost-effectiveness, data management capabilities, and security features, which have been positively appraised in prior qualitative research [40]; known limitations of videoconferencing in qualitative research (eg, technical issues and partial loss of nonverbal cues) were considered acceptable given the nature of the study topic. The interview guide (Multimedia Appendix 1) was developed iteratively by the research team and included open-ended questions about therapists’ experiences before, during, and after remote goal-setting encounters, including perceived advantages and barriers, adaptation strategies, and impacts on the therapeutic relationship. The guide was used flexibly rather than as a fixed script: questions served as points of departure for a conversation around each topic, the order of questions varied with the flow of each interview, and follow-up probes were used to invite elaboration, while the interviewer ensured that all topic areas were covered in every interview. The interview guide was not formally pilot tested; however, it was reviewed and refined through team discussion among the coauthors, who have established expertise in qualitative research methods and in rehabilitation goal setting. All interviews were video-recorded with participants’ permission. No one other than the interviewer and the participant was present during the interviews, and no repeat interviews were conducted; each participant was interviewed once. Formal field notes were not maintained during or immediately after data collection. Instead, the interviewer (YO) reviewed each video recording in full against the corresponding transcript on multiple occasions during the familiarization phase and recorded reflective observations and interpretive notes in the audit trail. For example, notes made during theme development recorded that therapists repeatedly described relying on partial or secondhand information (eg, answers relayed only as “probably...”), and these notes informed the interpretation, developed in theme 1, that remote delivery reduced therapists’ confidence in their clinical judgments.

Transcripts were produced in Japanese using AI-assisted first-pass transcription (Notta), after which YO manually reviewed each recording in full and corrected any errors against the audio and video. Analysis was conducted on the original Japanese transcripts to preserve contextual and cultural meaning. Illustrative quotations presented in this manuscript were translated from Japanese into English with the assistance of generative AI tools as a first-pass aid (see the Acknowledgments section), and were subsequently reviewed and corrected by the corresponding author (YO), a native Japanese speaker fluent in English, against the original Japanese audio and transcripts. Where culturally specific expressions had no direct English equivalent, the translation prioritized conceptual meaning over literal rendering, with the source Japanese preserved in the audit trail for transparency. Demographic and professional background information was collected through a brief online questionnaire covering profession, age, years of therapist experience, years of remote service experience, client age groups served, and broad diagnostic areas served remotely.

### Data Analysis

Data were analyzed using RTA as articulated by Braun and Clarke [30,31]. The analysis was primarily inductive and data-driven, and no predetermined coding framework was applied. Coding stayed close to the explicit, semantic content of participants’ accounts; more interpretive analysis was introduced in the later phases, for example, when accounts of hesitancy and reduced confidence were interpreted together as a disruption of professional certainty (theme 1). The reflective observations and interpretive notes described above contributed to this interpretive work. YO led all analytic phases. In phase 1 (familiarization), YO read each transcript repeatedly while checking it against the interview recordings. In phase 2 (initial coding), YO generated codes across the dataset, primarily at the semantic level. In phase 3 (candidate theme generation), codes were grouped to identify broader patterns of meaning and construct candidate themes. In phase 4 (theme review), candidate themes were reviewed against both the coded extracts and the full dataset for internal coherence and external distinctiveness. In phase 5 (theme definition and naming), themes were refined and named to capture the central organizing concept of each. In phase 6 (report production), the analytic narrative was developed by selecting illustrative quotations and relating the final themes back to the study aim [41]. Analytic materials were shared with all authors throughout the process; discussions were used to challenge interpretations and refine thematic boundaries rather than to establish coder agreement, consistent with RTA. Data were managed using Microsoft Excel and Microsoft Word; no dedicated qualitative data analysis software was used, as the dataset comprised 9 transcripts and the audit trail was maintained in a shared spreadsheet that all authors could access directly, preserving the traceability of coding decisions.

### Rigor and Trustworthiness

Trustworthiness was addressed through the criteria of credibility, transferability, dependability, and confirmability, following Nowell and colleagues’ [42] application of these criteria to thematic analysis. Credibility refers to the confidence that can be placed in the fit between participants’ accounts and the researchers’ representation of them. Credibility was strengthened through prolonged engagement with the data, including repeated reading of transcripts alongside audio recordings, collaborative analytic discussions among the 3 authors, attention to divergent and less common cases, and retention of the original Japanese data alongside English translations [33,35]. Transferability refers to the extent to which findings may be applicable in other settings, which readers can judge only if the study context is described in sufficient detail. Transferability was supported by detailed reporting of the study setting, participant characteristics, and analytic decisions, as presented in Table 1 and the Methods section [33]. Dependability refers to whether the research process is logical, traceable, and clearly documented. Dependability was enhanced through an audit trail documenting analytic decisions across the 6 phases of analysis, maintained in a shared spreadsheet [34]. Confirmability refers to the demonstration that interpretations are clearly derived from the data rather than from researcher predisposition alone. Confirmability was supported by this audit trail, team review of the links between data extracts and final themes, and reflexive consideration of the research team’s positioning, as described in the Research Team and Reflexivity section [34,35]. Participant transcript review, or member checking, was not undertaken. This decision was consistent with the study’s contextualist stance, which treats meaning as co-constructed through the analytic process rather than as something to be verified retrospectively by participants [31,35].

## Results

### Overview

Four themes were constructed that described therapists’ experiences of remote goal setting in rehabilitation: (1) losing touch: uncertainty in remote assessment and goal setting; (2) from direct intervention to coaching-oriented goal setting; (3) at home, at ease: needs that surface in clients’ own environments; and (4) when continuity becomes the goal. Each theme is described in turn below, and the relationships among the themes are then summarized. Table 2 summarizes each theme and the number of participants whose accounts contributed to it. Within-theme variation was retained in the analysis through close attention to less prominent perspectives and is reflected in the selection of quotations across participants; no minor themes or divergent cases inconsistent with the 4 main themes were identified that warranted construction as a separate theme.

**Table 2.** Themes, central organizing concepts, and participant contributions (N=9)^a^.

Theme	Central organizing concept	Participants contributing (n/9)
1. Losing touch: uncertainty in remote assessment and goal setting	Loss of embodied and contextual information disrupts clinical certainty.	9/9
2. From direct intervention to coaching-oriented goal setting	Remote constraints prompt a shift from provider to facilitator/coach.	8/9
3. At home, at ease: needs that surface in clients’ own environments	Home-based interaction puts clients at ease and discloses context and needs less visibility in clinic settings.	6/9
4. When continuity becomes the goal	Goals are reoriented toward continuity through ongoing monitoring, structured follow-up, and prevention of decline.	6/9

a Counts are based on at least one substantive contribution to the theme; counts do not imply quantification of prevalence.

### Theme 1: Losing Touch: Uncertainty in Remote Assessment and Goal Setting

The first theme captured therapists’ experiences of uncertainty in assessment and goal setting when working remotely. All 9 participants described how the inability to physically touch clients and the reduced access to nonverbal and contextual information limited their confidence in understanding clients’ conditions, needs, and priorities. In this study, “reduced visibility” did not mean that clients’ homes were invisible; rather, what therapists could see was restricted to the fragment of the environment and the body captured within the camera frame, shaped by camera angle, device placement, connection quality, and what the client or another person was able or willing to show. Unlike an in-person encounter, in which therapists could walk around, change their vantage point, and observe the whole body and setting, remote observation was partial and fixed. Therapists highlighted limitations in physical assessment and intervention, particularly in situations in which whole-body observation or hands-on assessment would normally inform clinical reasoning. One therapist stated, “It is difficult to understand everyday life scenes remotely...it is hard to picture daily life, or to imagine the actual situation just by listening” [Occupational therapist, participant 9]. Another reflected on the limits of remote postural assessment, explaining that a client’s sitting posture might look acceptable during a brief observation on screen but deteriorate over a longer period, something that could be detected only through sustained in-person observation: “With postural adjustment, there are aspects you only understand if you take time. It may seem to be going well in the moment, but posture can collapse over time, so you really need to observe it in person to know” [Occupational therapist, participant 8].

Therapists mentioned difficulty interpreting pauses, atmosphere, and other nonverbal features of interaction that would typically shape in-person communication. In addition, some felt constrained by having to depend on information mediated through third parties. In the Japanese service contexts described by participants, these third parties included, for older clients, care managers (care coordinators who arrange services under Japan’s long-term care insurance system) and visiting care staff, and, for children, parents and teachers who acted as the child’s spokespersons in remote consultations. Information about the client’s wishes then reached the therapist secondhand, already filtered through another person’s interpretation. This limited their ability to directly understand clients’ perspectives and the reasons underlying their preferences and behaviors. One therapist, who was supporting an older client with Parkinson disease, received information about the client’s wishes mainly through the care manager and care staff involved in the client’s daily care. She found that these relayed answers were often vague, which made it difficult to identify acceptable alternatives to the client’s stated wish. She explained, “I want to know why they want to do it that way, their particular preference, or their true feelings. Without that...it would really have been better if I could have directly asked the person themselves” [Occupational therapist, participant 7].

These accounts described more than practical inconvenience. Without touch and whole-body observation, assessments that would ordinarily feel routine became tentative, therapists made proposals by inference from secondhand information, and one therapist noted that whether a suggestion had worked often became clear only at the following session. In their accounts, information that therapists usually gather through vision, touch, and the atmosphere of an encounter had supported the confidence of their goal setting. Remote goal setting therefore involved interpretive uncertainty: a reduction in therapists’ professional certainty that they worked to manage through the adaptations described in the following themes.

### Theme 2: From Direct Intervention to Coaching-Oriented Goal Setting

The second theme, reported by 8 of the 9 participants, reflected a shift in therapists’ roles from directly intervening in clients’ bodies and activities to supporting goal setting through coaching. In this theme, “coaching-oriented” refers to the interactional role therapists adopted: facilitating clients’ reflection, self-monitoring, and problem-solving rather than delivering hands-on intervention. It is related to, but not the same as, client-centeredness (see Introduction): client-centeredness concerns whose priorities guide care, whereas coaching describes how therapists worked with clients when hands-on guidance was unavailable. For these therapists, coaching became a principal means of conducting client-centered goal setting remotely. Therapists described compensating for the limitations of remote care by gathering information in advance, using visual materials, and encouraging clients and family members to take a more active role in implementing strategies in daily life. Rather than positioning themselves primarily as direct providers of intervention, therapists described working more as partners who facilitated reflection, supported self-monitoring, and collaboratively developed practical strategies with clients and families.

Several therapists reported using self-monitoring tools, including pedometers and check sheets, to monitor clients’ physical activity levels and daily routines between sessions. Remote sessions gave therapists only a brief and delayed view of clients’ daily lives, and self-monitoring tools extended assessment and goal setting beyond the session itself: they supported clients’ reflection on their activity patterns and gave therapists more structured information about clients’ everyday activities, which informed subsequent goal-setting discussions. For example, one hospital-based therapist reviewed a weekly step count from a loaned pedometer with each client and negotiated a graded target for the following week. Notably, several therapists described these strategies not merely as substitutes for in-person methods but as improvements they valued in their own right: one explained that in conventional practice his advice would have ended in the session, whereas the online tools allowed him to follow clients’ behavior change between sessions, and another described slide-supported goal setting as something good “in its own way” that was unique to remote delivery.

Others used visual materials, including slide summaries and submitted videos, to overcome what they described as the limitations of verbal communication alone. One therapist, who had noticed in earlier group-based remote sessions that sharing slides on screen helped conversations run smoothly, applied this to individual goal setting with a young school-aged client: rating cards from a goal-setting tool were embedded in the slides, and the client’s own words were typed into the slides during the session and summarized visually, so that therapist and client were, in effect, looking at a shared record of the conversation as it developed: “I input the client’s narrative directly into the slides and summarised it for them, and by doing that, the conversation became smoother” [Occupational therapist, participant 6]. Physical activities such as skipping rope and using a horizontal bar could not be practiced live through the screen, so the same therapist treated them as a home program: the online sessions were used to agree on practical strategies the child would try at home, the parent recorded videos of the practice and sent them for monitoring, and the child reported back at the next session on which strategies had worked: “When practising tasks at home on a task-based approach, we discussed online what kinds of strategies could be used” [Occupational therapist, participant 6].

Family members and other supporters occupied a distinctive position in these remote encounters. Particularly for children, a parent or teacher was often physically present with the client while the therapist was not: they operated the device, recorded and sent videos, and carried out with the client the activities that the therapist would ordinarily have demonstrated in person. Therapists therefore had to put into words, and coach others through, actions they would normally have shown directly; as one therapist described, instead of demonstrating a technique and gradually fading support, she had to verbalize it for the mother to understand and carry out, which she found demanding. These arrangements created new demands, but they also distributed ownership of implementation across the therapist, the client, and the people around them. These adaptations nevertheless appeared to support a more collaborative form of goal setting, in which clients and families were encouraged to identify and monitor problems in their own daily lives. One therapist supported a middle-aged client who had returned to work following a neuromuscular condition and who completed an occupational balance check sheet several times a week and emailed it to him between weekly video sessions; the sheet gave the client herself a structured way of noticing and reporting what mattered: “Because they filled in the occupational balance check sheet, they always told me or reported what they, as the person concerned, thought was a problem that week” [Occupational therapist, participant 1].

### Theme 3: At Home, at Ease: Needs That Surface in Clients’ Own Environments

The third theme, raised by 6 of the 9 participants, described how remote sessions conducted in clients’ own living environments changed the goal-setting encounter in 2 intertwined ways: clients often appeared more at ease, and their everyday needs and circumstances became more visible to therapists. Therapists suggested that remote rehabilitation was not only a way of overcoming physical distance, but also a way of shifting the interaction from the clinician’s setting to the client’s own space. This appeared to allow clients to participate in a more relaxed and natural manner and enabled therapists to observe aspects of clients’ everyday environments that were not readily available in clinic-based encounters. Therapists described how being at home could reduce tension and make it easier for clients to disclose concerns and preferences. One therapist, whose clients often joined video consultations while working from home, noted, “Because the user could participate in a relaxed state, I felt as a therapist that it was easier to draw out their needs in a more natural way” [Physical therapist, participant 3]. Everyday remote contact could also create natural openings for goal-related conversation. Another therapist, who kept in regular video contact with a community-dwelling client, described how casual exchanges about the previous evening became shared topics that could be raised naturally in later sessions, weaving needs identification into ordinary conversation rather than formal questioning [Occupational therapist, participant 5].

Therapists also reported that remote sessions allowed them to directly observe everyday environments, such as desk setups, room layouts, and family situations, which supported more concrete assessment and goal setting. As one participant stated, “You can also see the desk environment here, and even the family situation is visible, so based on that, we can immediately check things and assess them” [Physical therapist, participant 3]. These accounts suggest that remote care sometimes enabled therapists to identify needs and priorities that may have been less visible in institutional settings. This visibility was of a different kind from the reduced visibility described in theme 1: relative to hands-on, whole-body assessment, remote observation remained partial, but relative to clinic-based encounters, in which the home is available only through the client’s description, the video call brought therapists directly into contact with the client’s everyday world.

### Theme 4: When Continuity Becomes the Goal

The fourth theme, articulated most explicitly by 6 of the 9 participants, concerned a shift in how therapists conceptualized the goals of rehabilitation in remote contexts. Faced with disrupted service access during the pandemic and with ongoing geographical or systemic barriers, therapists described reorienting goals away from functional improvement alone and toward maintaining continuity, ongoing monitoring of well-being, functional status, and daily engagement. Remote rehabilitation was often positioned as a means of sustaining structured follow-up and preventing decline or disengagement from services, rather than as a direct substitute for intensive physical intervention. In some cases, this monitoring function also carried an important relational dimension, as regular remote contact provided psychological reassurance and helped prevent social isolation. Some emphasized the importance of continuity across service transitions and the potential for remote follow-up to support self-management, including pain management. One hospital-based therapist, whose older clients returned to rural areas with limited transport after discharge, continued the hospital’s activity program through weekly video sessions and described the rationale for this follow-up: “By continuing the same kind of service remotely even after discharge, [we wondered] whether the onset of chronic pain could be prevented” [Physical therapist, participant 4]. He also noted that clients reported feeling reassured that support did not simply stop at discharge.

Others described the therapeutic value of remaining connected to clients over time. One therapist made regular evening video calls to a client with a psychosocial disability who had recently begun living alone and who was at risk of self-harm driven by loneliness and self-blame. He deliberately avoided making goals explicit with this client, because putting them into words risked drawing the client’s attention to questions of life and death; instead, the everyday contact itself carried the therapeutic purpose, oriented toward the client feeling that their life had worth and that tomorrow was worth reaching: “Rather than putting it into words or things like that, what I wanted them to feel was that it is okay to be alive. That became our goal, or rather our purpose” [Occupational therapist, participant 5]. A further participant, a pediatric therapist practicing in a region with many underserved communities who had heard of families relocating simply to access developmental services, emphasized equity of access, stating, “I wish people could receive services no matter where they live...I want to make sure that is guaranteed for them” [Occupational therapist, participant 2].

This reframing did not appear in therapists’ accounts as an abandonment of functional goals. Therapists continued to pursue functional gains where feasible through coached self-management (theme 2), while the immediate purpose of the remote encounter became sustaining engagement, monitoring change, and preserving the therapeutic relationship until more intensive intervention was possible or necessary. Continuity was described as an interim, complementary, or sometimes intrinsically meaningful goal, consistent with a client-centered orientation. The accounts also marked a clinical boundary: goals requiring hands-on assessment or intervention could not be fully pursued remotely and required a hybrid or later in-person response. These accounts indicate that, in remote contexts, therapists sometimes redefined the purpose of goal setting to include ongoing monitoring, sustained support, and continuity of care.

### Relationships Among Themes

**Figure 1.**
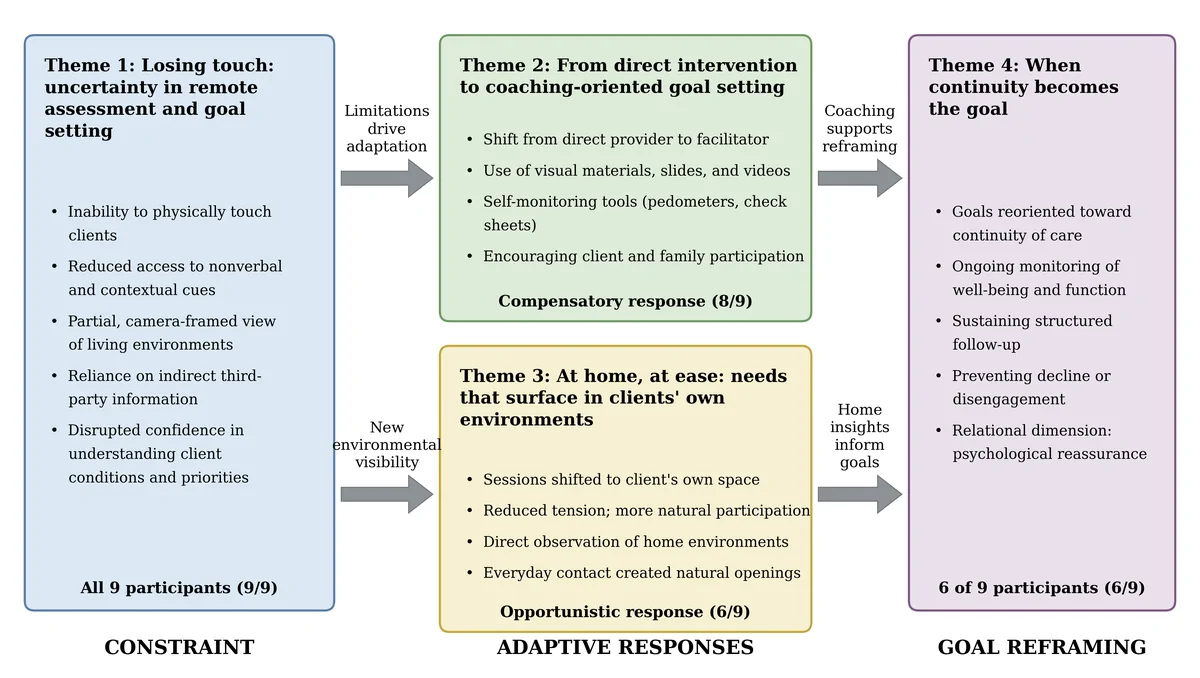
The authors’ interpretation of the relationships among the four themes: constraint (theme 1), adaptive responses (themes 2 and 3), and goal reframing (theme 4).

We interpreted the 4 themes as interconnected rather than independent. In our reading of the data, the constraints described in theme 1 formed the background against which 2 adaptive responses were described: a compensatory shift toward coaching-oriented goal setting (theme 2) and a recognition of needs made visible in clients’ home environments (theme 3). These responses, in turn, informed a broader reframing of the purpose of goal setting toward continuity and ongoing monitoring (theme 4). Figure 1 presents this interpretation of the relationships among the themes; it summarizes our analytic account rather than an observed temporal or causal sequence.

## Discussion

### Principal Findings

In these therapists’ accounts, remote goal setting changed both the information available for clinical judgment and the purposes assigned to rehabilitation goals; it was described as a distinct form of practice shaped by constraint and possibility rather than as a diminished copy of in-person goal setting. This study examined the goal-setting experiences of Japanese therapists who practiced telerehabilitation when remote service delivery was not yet routine, frequently under pandemic-related pressure. As summarized in Figure 1, the constraints described in theme 1 were met with 2 adaptive responses, a shift toward coaching-oriented practice (theme 2) and attention to needs visible in clients’ home environments (theme 3), and these together informed a reframing of goals toward continuity and ongoing monitoring (theme 4). Reduced visibility and the inability to touch introduced uncertainty into assessment and interpretation, yet remote care also appeared to create opportunities for collaborative and contextually grounded goal setting. Our analysis suggests that the conduct of remote goal setting may depend on how telerehabilitation encounters are prepared, structured, and supported, and not on access to technology alone; this interpretation, derived from the present data, is consistent with the broader telehealth implementation literature [43,44]. This is relevant given the growing recognition of telerehabilitation as a means of supporting physical activity and preventing functional decline among populations with limited access to conventional rehabilitation, including frail older adults [6,7].

### Comparison With Prior Work

The first theme, losing touch, is broadly consistent with previous literature showing that telerehabilitation can limit physical assessment and reduce access to nonverbal and contextual cues [45,46]. However, this study extends that literature by suggesting that these constraints may be experienced not merely as practical barriers but as a disruption to therapists’ professional certainty, as described in the final paragraph of theme 1. In other words, remote care appeared to introduce interpretive uncertainty, defined as uncertainty in making clinical sense of partial or mediated information, by reducing the embodied and contextual information on which therapists often rely, sometimes implicitly, to make sense of clients’ needs, priorities, and readiness for goal setting [16,47]. In this sample, the challenge of remote goal setting was therefore not simply that it is “harder through a screen,” but that it could alter the foundations of clinical reasoning and confidence in goal negotiation.

The second theme, from direct intervention to coaching-oriented goal setting, also aligns with prior work suggesting that telerehabilitation can shift therapists’ roles from hands-on intervention toward facilitation, coaching, and collaborative problem-solving [48,49]. In the present study, therapists described using advanced information gathering, visual tools, and self-monitoring resources to compensate for the limits of remote interaction. In particular, pedometers and activity check sheets were used to monitor clients’ physical activity between sessions. This aligns with recent evidence suggesting that wearable activity trackers and structured self-monitoring tools may contribute to improvements in physical function among frail older adults, although their effects on overall daily physical activity levels remain inconclusive [50,51]. These adaptations did not appear to function only as substitutes for in-person practice. Rather, they sometimes created a more co-constructed form of goal setting by encouraging clients and families to take a more active role in identifying priorities, monitoring difficulties, and implementing strategies in daily life. This connects directly to the calls, outlined in the Introduction, for more collaborative and client-centered goal setting in Japanese rehabilitation [13,14,21]: coaching, in these accounts, was the interactional form through which client-centered principles were enacted when hands-on guidance was unavailable. Coaching should not, however, be equated with client-centeredness: remote delivery does not automatically make practice client-centered, which continues to depend on clients genuinely owning and negotiating their goals. When adequately supported, the constraints of remote delivery may create opportunities for more participatory and, thereby, more client-centered, goal-setting practices.

The third theme, at home, at ease, highlights a potential advantage of remote care. Previous studies have suggested that, by situating rehabilitation within the client’s everyday environment, telerehabilitation can make assessment and goal setting more closely reflect clients’ actual daily lives [52,53]. The present findings add that the value of home-based interaction may lie in moving the encounter from the clinician’s setting to the client’s own space, as well as in observing the physical environment. Therapists perceived that clients were often more relaxed at home, and this appeared to make it easier for some clients to express concerns, preferences, and personally meaningful goals. In this sample, remote goal setting sometimes appeared to make everyday needs more visible both because parts of the home environment could be observed directly and because clients seemed more comfortable in their own surroundings [54].

The fourth theme, when continuity becomes the goal, suggests that the goals of rehabilitation may be repositioned in remote contexts. Rather than focusing solely on functional improvement, therapists described reorienting goals toward maintaining continuity through ongoing monitoring of well-being, functional status, and daily engagement. Remote rehabilitation was often positioned as a means of sustaining structured follow-up and preventing decline or disengagement from services. In some cases, this monitoring function also carried an important relational dimension, as regular remote contact provided psychological reassurance and helped prevent social isolation. This should not be interpreted as a retreat from rehabilitation goals, but rather as a clinically meaningful repositioning of goals under specific service constraints. Such a shift may be particularly relevant in chronic-condition management and longer-term community-based rehabilitation, where self-management, continuity, and therapeutic connection are often central to meaningful care [55,56]. In frailty management, this may be especially important, as sustained physical activity and ongoing monitoring are needed to help prevent further decline. Remote goal setting and digital tracking may provide a practical way to maintain therapeutic engagement and support regular activity beyond scheduled rehabilitation sessions [27,50,51].

### Implications for Telerehabilitation Design, Implementation, and Practice

These findings have several implications for the design, implementation, and delivery of telerehabilitation. Remote delivery of rehabilitation, including its goal-setting component, should not be assumed to be suitable for every client, clinical purpose, or stage of care. Rather, decisions about when goal setting is conducted remotely, in person, or in combination need to consider client characteristics, family support, communication needs, home and service environments, and the type of clinical judgment required. The findings also suggest that remote goal setting benefits from structured preparation. Collection of videos, check sheets, or contextual information may help compensate for therapists’ reduced access to embodied and situational cues; this implication is consistent with prior work on telehealth-based goal setting and goal management [57]. In this process, families and support persons may need to be positioned not simply as assistants, but as active collaborators whose roles and whose influence on the client’s own voice are explicitly negotiated. Visual tools, self-monitoring resources, and shared documentation may also support shared understanding and continuity between sessions. For therapists, this suggests a need for telerehabilitation-specific training that extends beyond technical competence to include rapport-building, verbal coaching, collaborative goal negotiation, and managing interpretive uncertainty; our findings align here with previous work on remote consultation skills [58].

The findings also support hybrid models in which remote goal setting is used selectively alongside in-person assessment or intervention. In-person review may remain important when hands-on assessment, detailed observation, or risk management is required: initial goal setting and physical assessment might take place in person, with goals then reviewed, monitored, and renegotiated remotely, and needs surfacing during remote sessions might prompt targeted in-person review. Because goal negotiation is an ongoing process embedded in the therapeutic alliance rather than a one-off event, hybrid delivery does not remove the need for remote goal-setting skills; it distributes goal-setting work across delivery modes according to clinical purpose. Organizations may therefore benefit from clear hybrid pathways that identify when remote goal setting is appropriate, what preparation is needed, and when in-person review should be prioritized.

### Strengths and Limitations

A strength of this study is that it qualitatively examined remote goal setting from the perspective of rehabilitation therapists practicing in Japan, a context that has received limited attention in the telerehabilitation literature. Participants were recruited from 9 different workplaces across metropolitan and regional areas of Japan, including hospital-based, private-practice, and community-based services. This reduces the likelihood that the patterns identified reflect the practices or culture of a single institution and supports modest transferability to other Japanese rehabilitation settings. Another strength is that the study captured therapists’ experiences across the phases before, during, and after remote goal-setting encounters. This enabled a broader understanding of how preparation, interaction, and follow-up shaped remote goal setting in practice. The analysis also identified not only barriers, but also potential advantages of remote goal setting, generating practical implications for service design and implementation.

The study also has limitations. The sample was small, included more occupational therapists (n=7) than physical therapists (n=2), and included more men (n=7) than women (n=2); the perspectives of physical therapists and of female therapists are therefore less fully represented, and the findings should not be read as a balanced account of Japanese rehabilitation practice. Although information power guided our judgment of the dataset, therapists who declined or were not reached may have experienced remote goal setting differently, and a different sample might have led to additional or different themes. The analysis is therefore presented as a contextually situated account of these 9 therapists’ experiences rather than an exhaustive description of remote goal setting in Japan. The findings are based on retrospective self-report rather than observation of practice. Session frequency and appointment duration were not collected systematically, which limits comparison with in-person service schedules. Participants represented selected clinical areas and all had some telerehabilitation experience, which may limit transferability to other contexts or to therapists without such experience. The study did not include clients’ or families’ perspectives and therefore captures only one side of the goal-setting process. Microsoft Excel and Word provided a transparent audit trail but fewer automated retrieval and version-control functions than dedicated qualitative data analysis software. Finally, because the data were collected during the COVID-19 pandemic, some findings may reflect pandemic-related service disruption as well as more enduring features of telerehabilitation.

### Future Research

Future research should examine remote goal setting from the perspectives of clients and families to better understand how shared understanding, comfort, participation, and collaboration are experienced across stakeholders. Further research is also needed on tools and supports designed specifically for remote goal setting, such as visual prompts, structured preparation tools, and shared documentation resources. Future studies should also evaluate hybrid models of goal setting that combine remote and in-person elements according to clinical purpose, client preference, and service context. Comparative research across health care systems and cultural contexts may help clarify how institutional, regulatory, and social factors shape remote goal setting in practice. Finally, while some implications of the present findings may be relevant to chronic-condition management and long-term community-based rehabilitation, further research is needed to examine whether similar patterns are observed across other rehabilitation populations and service models.

### Conclusions

This qualitative study showed that therapists experienced remote goal setting as both constraining and enabling. Reduced visibility and the inability to touch created uncertainty in assessment and interpretation, yet remote encounters also supported new forms of collaboration, greater visibility of clients’ everyday contexts, and a reorientation of goals toward continuity and ongoing monitoring. Remote goal setting should therefore not be viewed simply as a lesser version of in-person rehabilitation. Rather, it represents a distinct form of practice that requires its own clinical reasoning, communication strategies, and implementation supports.

## Supplementary material

10.2196/101355 Multimedia Appendix 1 Interview guide (translated from Japanese to English).

10.2196/101355 Checklist 1 COREQ checklist.

## Abbreviations

COREQ: Consolidated Criteria for Reporting Qualitative Research
MOT: Master of Occupational Therapy
RTA: reflexive thematic analysis
