# Supplementary material for: Goal Setting During Telerehabilitation in Japan: Qualitative Study of Occupational Therapists’ and Physical Therapists’ Experiences Using Reflexive Thematic Analysis
Source: JMIR Rehabil Assist Technol. 2026 Aug 21;13:e101355. doi: 10.2196/101355 (PMC13496387; doi:10.2196/101355)
Supplement: Supplementary file 2 [file rehab-v13-e101355-s001.docx]

**Interview Guide (Translated from Japanese to English)**

**(1) Before Goal Setting**

1. Please tell me what prompted you to conduct goal setting, or to ask about the needs of the client/patient, remotely.
2. Please tell me what preparations you made when conducting goal setting, or when asking about the needs of the client/patient, remotely.
3. Did you receive any training before providing services remotely? If so, what kind of training was it?
4. Did you feel confident in carrying this out? Why or why not?
5. Please tell me what resources you used when conducting goal setting, or when asking about the needs of the person concerned, remotely.
6. How did you obtain those resources?
7. Who covered the costs associated with those resources?
8. Did you experience any difficulties in obtaining those resources?

**(2) Goal Setting**

1. How did you conduct goal setting, or ask about the needs of the client/patient, remotely?
2. Please tell me who was actually present at the time. Also, if each person had a specific role, please describe their role.
3. How long did it take?
4. Did you experience any difficulties when conducting goal setting, or when asking about the needs of the person concerned, remotely?

**(3) After Goal Setting**

1. After conducting goal setting, or asking about the needs of the client/patient, remotely, what kind of intervention did you provide, and how did you provide it?
2. After that, were you able to achieve the goal or meet the identified needs? Why or why not?

This concludes the interview. Thank you very much for your participation.

**Yuho Okita**
Research Collaborator
Graduate School of Biomedical Sciences
Nagasaki University
